# φX216, a P2-like bacteriophage with broad *Burkholderia pseudomallei* and *B*. *mallei* strain infectivity

**DOI:** 10.1186/1471-2180-12-289

**Published:** 2012-12-07

**Authors:** Brian H Kvitko, Christopher R Cox, David DeShazer, Shannon L Johnson, Kent J Voorhees, Herbert P Schweizer

**Affiliations:** 1 Department of Microbiology, Immunology and Pathology, Colorado State University, IDRC at Foothills Campus, Fort Collins, CO, 80523-0922, USA; 2 Department of Chemistry and Geochemistry, Colorado School of Mines, Golden, CO, USA; 3 United States Army Medical Research Institute of Infectious Diseases, Fort Detrick, MD, USA; 4 Los Alamos National Laboratory Genome Science Group, Joint Genomics Institute, Los Alamos, NM, USA

**Keywords:** Bacteriophage, *Burkholderia pseudomallei*, *B*. *mallei*, P2, Prophage distribution, Phage-based diagnostics

## Abstract

**Background:**

*Burkholderia pseudomallei* and *B*. *mallei* are closely related Category B Select Agents of bioterrorism and the causative agents of the diseases melioidosis and glanders, respectively. Rapid phage-based diagnostic tools would greatly benefit early recognition and treatment of these diseases. There is extensive strain-to-strain variation in *B*. *pseudomallei* genome content due in part to the presence or absence of integrated prophages. Several phages have previously been isolated from *B*. *pseudomallei* lysogens, for example φK96243, φ1026b and φ52237.

**Results:**

We have isolated a P2-like bacteriophage, φX216, which infects 78% of all *B*. *pseudomallei* strains tested. φX216 also infects *B*. *mallei*, but not other *Burkholderia* species, including the closely related *B*. *thailandensis* and *B*. *oklahomensis*. The nature of the φX216 host receptor remains unclear but evidence indicates that in *B*. *mallei* φX216 uses lipopolysaccharide O-antigen but a different receptor in *B*. *pseudomallei*. The 37,637 bp genome of φX216 encodes 47 predicted open reading frames and shares 99.8% pairwise identity and an identical strain host range with bacteriophage φ52237. Closely related P2-like prophages appear to be widely distributed among *B*. *pseudomallei* strains but both φX216 and φ52237 readily infect prophage carrying strains.

**Conclusions:**

The broad strain infectivity and high specificity for *B*. *pseudomallei* and *B*. *mallei* indicate that φX216 will provide a good platform for the development of phage-based diagnostics for these bacteria.

## Introduction

*Burkholderia pseudomallei* and *B*. *mallei* are facultative intracellular Gram-negative human and animal pathogens and the causative agents of the endemic diseases melioidosis and glanders, respectively [1-4]. Because of their intrinsic antibiotic resistance and high mortality caused by the respective diseases despite aggressive treatment, *B*. *pseudomallei* and *B*. *mallei* are classed as Category B Select Agents of bioterrorism. *B*. *pseudomallei* is a ubiquitous Gram-negative soil bacterium endemic to southeast Asia and northern Australia and possesses a genome showing extensive strain-to-strain variation. A significant portion of this genome variation is due to the presence or absence of integrated prophages [5-7]. *B*. *pseudomallei* strains commonly carry at least one integrated prophage and multiple phages have been isolated from lysogenic *B*. *pseudomallei* strains [8-10]. *B*. *mallei*, on the other hand, exists in a zoonotic reservoir and appears to have evolved from *B*. *pseudomallei* by genome reduction [11]. Previously sequenced *B*. *mallei* strains do not carry intact prophages but can be infected by many phages isolated from *B*. *pseudomallei*[8-10,12].

In this study we isolated φX216 from spontaneous plaques formed by the Thai *B*. *pseudomallei* environmental isolate E0237 and determined its DNA sequence. φX216 is a member of the widely distributed *Burkholderia* P2-like phage family [8]. It has broad *B*. *pseudomallei* strain infectivity for members of the *B*. *pseudomallei* clade. Our data indicate that φX216 may serve as a good candidate for developing rapid phage-based diagnostic tools for *B*. *pseudomallei* and *B*. *mallei*.

## Results and discussion

### ϕX216 isolation and host range

**Figure 1:**
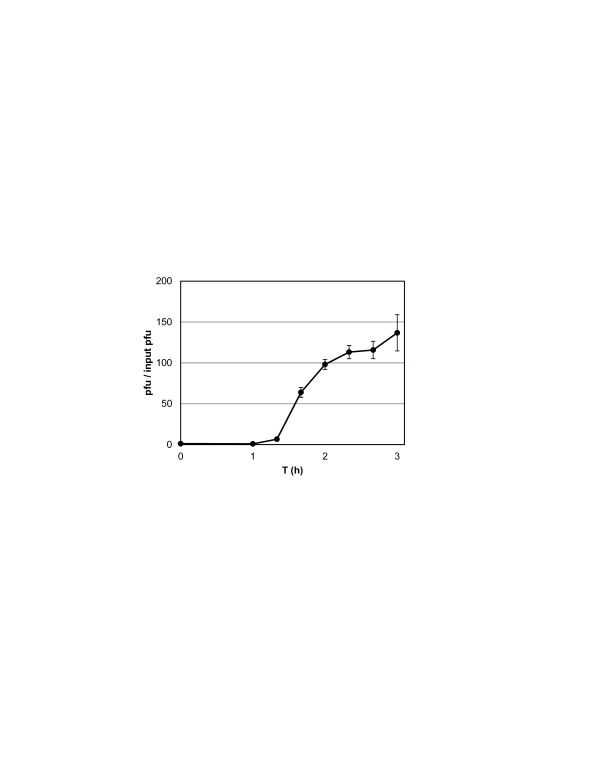
**φX216 one**-**step growth curve.** φX216 was adsorbed to *B*. *mallei* ATCC23344 cells for 15 min, inoculated into LB + 2% glycerol, and cultures were incubated at 37°C with shaking. Triplicate aliquots were removed at the indicated time intervals and used to inoculate plaque plates to determine pfu/mL. The pfu/mL values were divided by the means of the T_0_ and T_1_ (1 h) phage concentrations to adjust to pfu/input pfu.

*B*. *pseudomallei* environmental isolate E0237 was observed to spontaneously form clear phage plaques after plating of overnight liquid cultures on agar plates. The spontaneously released phage, φX216 (named for the E0237 laboratory stock number), was plaque purified on *B*. *pseudomallei* strain 2698a and used to create medium-titer [10^6^ plaque forming units (pfu)/mL] plate lysates with a variety of *B*. *pseudomallei* host strains and high-titer (10^8^ pfu/mL) liquid lysates using *B*. *mallei* ATCC23344. This strain was also chosen for production of larger volume liquid lysates to prevent contamination with other phages as it is not predicted to contain a prophage [8]. One-step growth curves demonstrated that φX216 has an approximate 60-minute latent phase, an 80-minute life cycle, and a burst size of 120 pfu per infected cell (Figure 1). φX216 formed plaques on 56 of a panel of 72 *B*. *pseudomallei* strains composed of 30 environmental and 30 clinical isolates from Thailand, as well as 12 well-characterized strains from various sources, some of which are commonly used laboratory strains (see Additional file 1). At 77.8%, φX216 has one of the broadest strain infectivity ranges reported for a *B*. *pseudomallei* phage, comparing favorably with the Thai soil phages ST2 (78%, 49/63) and ST96 (67%, 42/63) [13,14]. φX216 plaques were 1–2 mm in diameter and mostly-clear on the majority of *B*. *pseudomallei* strains although there was some strain-dependent variation in plaque appearance with some forming pinpoint and/or turbid plaques. In addition, φX216 was also able to form plaques on all (9/9) *B*. *mallei* strains tested. In contrast, φX216 did not form plaques on closely related (*B*. *thailandensis* and *B*. *oklahomensis*) or other (*B*. *ubonenesis*, *B*. *vietnamensis* and *B*. *gladioli* pathovar *cocovenenans*) *Burkholderia* species (see Additional file 1). Although fewer isolates of these species were tested, φX216 does appear to have specificity for *B*. *pseudomallei* and *B*. *mallei* as compared with ST2 and ST96, which formed plaques on five of seven tested *B*. *thailandensis* strains. Because of the close relatedness of *B*. *pseudomallei* and *B*. *thailandensis* it will be prudent to assess more *B*. *thailandensis* strains as they become available to further support the claim of *B*. *pseudomallei* specificity.

Of the 56 *B*. *pseudomallei* strains that could be infected with φX216, 24 showed decreased relative plaquing efficiencies with the *B*. *mallei* lysate. However, when φX216 lysates were propagated two to three times on these initially low plaquing efficiency strains, lysates were obtained that then plaqued with titers of of 10^5^ to 10^6^ pfu/mL on those same strains. The reason(s) for low plaquing efficiencies of *B*. *mallei* lysates on some *B*. *pseudomallei* strains remain unclear but probably reflect some kind of host restrictive mechanism(s).

### ϕX216 host receptor

Experiments with *B*. *mallei* host strains indicated that *B*. *pseudomallei* phages φ1026b, φK96243 and φE202 use the lipopolysaccharide (LPS) O-antigen as a host receptor [8-10]. *B*. *mallei* O-antigen mutants cannot support infection by these phages and infection is restored if the O-antigen mutation is complemented. φX216 is also unable to infect *B*. *mallei* O-antigen mutants but, surprisingly, infection is not restored by complementing the mutation (see Additional file 1). As opposed to *B*. *mallei*, *B*. *pseudomallei* O-antigen mutants still support infection by φX216. Both an engineered deletion of the *wbiE* gene in *B*. *pseudomallei* Bp82 as well as 10 mapped transposon insertions in the *wbi* genes of *B*. *pseudomallei* 1026b formed φX216 plaques with an efficiency comparable to their respective parent strains. Therefore, φX216 may use the wild-type *B*. *mallei* O-antigen as a host receptor but not in *B*. *pseudomallei* where it uses a different receptor that is absent from *B*. *mallei*[11].

### ϕX216 genome characterization and chromosomal attachment site

**Figure 2:**
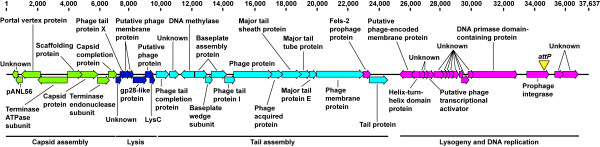
**φX216 genome annotation.** Gene clusters and their predicted functions are indicated in different colors. Predicted capsid structural and assembly genes are shown in lime, host lysis proteins are shown in blue, genes required for phage tail structure and assembly are shown in cyan, and genes encoding proteins involved in lysogeny and DNA replication are shown in magenta. The phage attachment site (*attP*) is indicated by a yellow triangle. Sequence numbering is shown above

To ascertain genomic features of φX216, we initially determined the entire φX216 genome sequence by low-coverage Sanger sequencing of plasmid clones generated by subcloning of φX216 DNA fragments and gap closing using sequence information obtained from PCR amplicons. This was supported by deep sequencing using the Illumina platform. Differences between Sanger and Illumina sequence runs were resolved by Sanger sequencing of specific phage DNA fragments obtained by PCR amplification using purified phage DNA and chromosomal DNA from φX216 lysogens as templates. The φX216 genome is 37,637 bases in length with a G + C content of 64.8% (GenBank: JX681814). GeneMark software predicted 47 open reading frames (Figure 2). The genome can be subdivided into predicted regions associated with capsid structure and assembly, host cell lysis, tail structure and assembly, and DNA replication and lysogeny (Figure 2). To determine the chromosomal attachment site, the φX216 lysogen Bp523 was isolated. Sequencing of the *attBattP* junction in this lysogen confirms the *attP* site of φX216 to be in the 3’ end of the predicted integrase gene corresponding to phage genome integration at tRNA-Phe (*attB*) [8].

Based on its genome sequence, φX216 is a P2-like member of the Myoviridae subgroup A. Its shares 99.8% pair-wise identity with φ52237 isolated from *B*. *pseudomallei* Pasteur 52237 (GenBank: DQ087285.2) [8]. There are 55 differences observed between φX216 and φ52237, which were independently confirmed by both Illumina and Sanger sequencing. The majority of these differences, cluster within a six gene region predicted to be associated with tail structure and assembly although only 14 are missense mutations resulting in amino acid alterations. However, these mutations are of no biological consequence since φ52237 and φX216 were found to have identical host ranges (see Additional file 1).

Illumina sequencing also produced a second 1,141-bp contig independent of the φX216 genome contig. This contig has 100% pairwise identity with the highly active IS*407a* insertion element found in the *B*. *mallei* genome [11]. At present we do not know whether this contig is the result of IS*407a* insertion in a sub-population of φX216 virions during preparation of the *B*. *mallei* lysates used for Illumina sequencing or an integral part of φX216 DNA. However, since the IS*407a* insertion was absent from the genome sequence obtained by Sanger sequencing it is unlikely an indigenous part of the φX216 genome.

### *Burkholderia* P2-like prophage distribution and correlation with ϕX216 host range

Although φX216 has a broad *B*. *pseudomallei* host range it fails to form plaques on approximately 22% of the strains tested in this study. We sought to determine if this was perhaps due to infection immunity conferred by the presence of related prophages.

**Figure 3:**
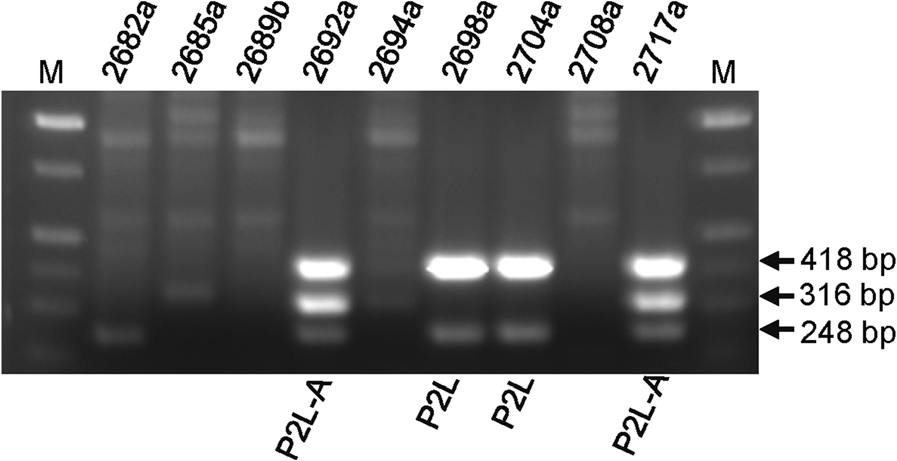
**Multiplex PCR for detection of φX216**-**related P2**-**like prophage in *B***. ***pseudomallei *strains.** Genomic DNA preparations of *B*. *pseudomallei* strains were used as PCR templates in multiplex PCR. Upper and lower fragments only (*B*. *pseudomallei* 2698a and 2704a) indicates presence of a P2-like (P2L) prophage. The presence of three fragments (*B*. *pseudomallei* 2692a and 2717a) indicates presence of a P2-like subgroup A prophage (P2L-A). The three marked DNA fragments correspond (top-to-bottom) to the fels-2 PCR product (418 bp), the *int* gene PCR product (316 bp), and the capsid gene N PCR product (248 bp). Lanes M, Hi-Lo molecular size ladder from Minnesota Molecular (Minneapolis, MN).

To that end, we designed a series of multiplex and individual PCR probes based on six isolated or predicted *Burkholderia* P2-like phages from Ronning *et al*. [8]. These included three subgroup A (φE202, φK96243 and φ52237/φX216) and three subgroup B (φE12-2, GI15, PI-E264-2) P2-like phages (see Additional file 2) [8]. PCR probes were designed to identify candidate P2-like prophages with increasing levels of relatedness to φX216/φ52237. The P2-like 1 and P2-like 2 probes amplify regions in the capsid gene (gene #6; for gene numbers see GenBank: JX681814) and Fels-2 gene (gene #29) and are conserved in both P2-like A and B subgroups. The P2-like subgroup A-specific probe amplifies in the integrase gene (gene #45). The φX216 scrnA and scrnB probes are specific to φX216/φ52237 and amplify DNA fragments from φX216 gene #46 and from the intergenic region between φX216 genes #30 and #31, respectively. The GI2 (Genomic island 2) probe amplifies the junction between the bacterial and prophage genomes at tRNA-Phe, predicted to serve as the *attB* site for *Burkholderi*a subgroup A phages [8,9]. We found that P2-like prophages are very common in *B*. *pseudomallei* strains (Table 1). Indeed, PCR analysis revealed that 30 out of 72 *B*. *pseudomallei* strains tested allowed amplification of DNA fragments indicative of the presence of a P2-like prophage (see Figure 3 for representative examples). Of those 30, 25 tested positive for subgroup A prophages. Six of those, including E0237, produced PCR results indicative of a close relationship with φ52237/φX216. *B*. *pseudomallei* 1710b, K96243, S13 and 1026b each produced PCR results that match sequence-based predictions for the presence of prophages [7,8,15]. Whereas strain 1710b is negative for a P2-like prophage, K96243 and S13 are both positive for subgroup A prophages (Table 1). Furthermore, 1026b is predicted to carry a φ52237-like prophage that is split into two fragments located in different regions of chromosome I (GenBank:CP002833.1, Locus # BP1026B_I0126- I0172 and BP1026B_ I3339-I3345). It is important to note that a positive hit for a subgroup A prophage does not exclude the possibility of a strain possessing multiple subgroup A prophages or more distantly related P2-like prophages. For instance, *B*. *pseudomallei* K96243 encodes both the φK96243 subgroup A prophage in genomic island 2, as well as the predicted subgroup B prophage GI15 on chromosome II, but the subgroup A PCR results hide the presence of the subgroup B GI15 prophage due to the fact that the GI15 probe amplicons are identical in size to those from the φK96243 prophage. The PCR probe results also do not indicate whether the candidate prophages can release viable phage progeny or are defective, as observed with the 1026b split φ52237-like prophage. The 30 strains that produced positive hits for P2-like prophages were additionally screened with the GI2 PCR probe. Strain 1710b was used as a P2-like-minus negative control. The 25 subgroup A candidate strains all produced positive PCR results for prophage integration into the 3’ end of the tRNA-Phe gene resulting in the formation of genomic island 2. The five candidates that failed to produce a positive GI2 PCR result were categorized as P2-like only. While our results do not definitively identify these five P2-like candidates as subgroup B members, subgroup B phages are predicted to use a different *attB* site and integration mechanism [8].

**Table 1:** ***B***. ***pseudomallei*P2**-**like prophage distribution screen**

		**P2**-**like prophage PCR probe results**					
		**Multiplex**					
***B***. ***pseudomallei***	**Candidate P2**-**like prophage group **^**a**^	**P2**-**like 1**	**P2**-**like 2**	**P2**-**like group A**	**φX216 scrnA**	**φX216 scrnB**	**GI2**
**Strains with high φX216 plaquing efficiency **^**b **^**66.7% (22/33**) **P2**-**like prophage candidate positive strains**							
2668a	φ52237-like	+	+	+	+	+	+
E0237 ^c^	φ52237-like	+	+	+	+	+	+
E0394	φ52237-like	+	+	+	+	+	+
1026b ^d^	φ52237-like	+	+	+	+	+	+
708a	φ52237-like ^e^	+	+	+	+	-	+
2618a	P2L-A	+	+	+	-	-	+
2661a	P2L-A	+	+	+	-	-	+
2692a	P2L-A	+	+	+	-	-	+
2717a	P2L-A	+	+	+	-	-	+
E0021	P2L-A	+	+	+	-	-	+
E0235	P2L-A	+	+	+	-	-	+
E0279	P2L-A	+	+	+	-	-	+
E0345	P2L-A	+	+	+	-	-	+
E0384	P2L-A	+	+	+	-	-	+
E0386	P2L-A	+	+	+	-	-	+
K96243 ^f^	P2L-A	+	+	+	-	-	+
S13 ^g^	P2L-A	+	+	+	-	-	+
2698a	P2L	+	+	-	-	-	-
2704a	P2L ^h^	+	+	-	-	+	-
E0342	P2L	+	+	-	-	-	-
E0366	P2L	+	+	-	-	-	-
E0377	P2L	+	+	-	-	-	-
2613a		-	-	-	-	-	ND ^i^
2667a		-	-	-	-	-	ND
2673a		-	-	-	-	-	ND
2682a		-	-	-	-	-	ND
2769a		-	-	-	-	-	ND
E0016		-	-	-	-	-	ND
E0034		-	-	-	-	-	ND
E0241		-	-	-	-	-	ND
E0356		-	-	-	-	-	ND
E0411		-	-	-	-	-	ND
MSHR305		-	-	-	-	-	ND
**Strains with low φX216 plaquing efficiency **^**j **^**17.4% (4/23), P2**-**like prophage candidate positive strains**							
2625a	φ52237-like	+	+	+	+	+	+
2670a	P2L-A	+	+	+	-	-	+
E0037	P2L-A	+	+	+	-	-	+
E0380	P2L-A	+	+	+	-	-	+
2637a		-	-	-	-	-	ND
2650a		-	-	-	-	-	ND
2660a		-	-	-	-	-	ND
2685a		-	-	-	-	-	ND
2708a		-	-	-	-	-	ND
2719a		-	-	-	-	-	ND
2764b		-	-	-	-	-	ND
E0024		-	-	-	-	-	ND
E0031		-	-	-	-	-	ND
E0181		-	-	-	-	-	ND
E0371		-	-	-	-	-	ND
E0372		-	-	-	-	-	ND
E0378		-	-	-	-	-	ND
E0383		-	-	-	-	-	ND
E0393		-	-	-	-	-	ND
1710a		-	-	-	-	-	ND
1710b ^k^		-	-	-	-	-	-
1106b		-	-	-	-	-	ND
406e		-	-	-	-	-	ND
**Non**-**φX216 plaquing strains 25.0% (4/16), P2**-**like prophage candidate positive strains**							
2671a	P2L-A	+	+	+	-	-	+
2674a	P2L-A	+	+	+	-	-	+
2677a	P2L-A	+	+	+	-	-	+
Pasteur 6068	P2L-A	+	+	+	-	-	+
2614a		-	-	-	-	-	ND
2617a		-	-	-	-	-	ND
2640a		-	-	-	-	-	ND
2665a		-	-	-	-	-	ND
2689b		-	-	-	-	-	ND
2694a		-	-	-	-	-	ND
E0008		-	-	-	-	-	ND
E0183		-	-	-	-	-	ND
E0350		-	-	-	-	-	ND
E0396		-	-	-	-	-	ND
1106a		-	-	-	-	-	ND
MSHR668		-	-	-	-	-	ND

^a^ φ52237-like assignment; positive PCR amplicons from multiplex probes P2-like 1, P2-like 2, P2-like group A, and individual PCR probes φX216 scrnA, φX216 scrnB and GI2. P2L-A assignment; positive PCR amplicons from multiplex probes P2-like 1, P2-like 2, P2-like group A and individual PCR probe GI2. P2L assignment; positive PCR amplicons from multiplex probes P2-like 1, P2-like 2.

^b^Confluent lysis when spot tested with ~10^6^ pfu φX216.

^c^φX216 source strain.

^d^1026b φ52237-like prophage is split into two segments and likely non-functional [15].

^e^P2-like prophage group cannot be determined based on PCR results. May be P2L-A or φ52237-like.

^f^φK96243 prophage (group P2-A) located at GI2 [9].

^g^Encodes the predicted prophage PI-S13-1 (group P2-A) [88].

^h^P2-like prophage group cannot be assigned based on PCR results. May be P2L or φ52237-like.

^I^ND, GI2 probe results not determined.

^j^Non-confluent lysis / individual plaques when spot tested with ~ 10^6^ pfu φX216.

^k^The strain 1710b genome does not contain a P2-like prophage or prophage insertion at GI2.

There is a strong correlation between P2-like prophage-positive *B*. *pseudomallei* strains and high efficiency plaquing by φX216 on those strains (specificity 79.5%, positive predicative value 73.3%). In other words, it seems as though many *B*. *pseudomallei* strains that can be efficiently infected by φX216 have been previously infected by one of its P2-like relatives and, strictly speaking, have been converted into lysogens.

## Conclusions

Phage φX216 has one of the highest strain infectivity rates reported among the *B*. *pseudomallei* phages characterized to date. Our results indicate that in contrast to previously isolated phages, φX216 infects and propagates only on strains belonging to the *B*. *pseudomallei* clade. This is a desirable diagnostic trait and we believe φX216 represents a good candidate platform for the development of phage-based *B*. *pseudomallei* diagnostic tools. Although φX216 infects both *B*. *pseudomallei* and *B*. *mallei*, these two species can be distinguished using φ1026b which is *B*. *mallei*-specific [10]. The independent isolation of nearly identical φX216 and φ52237 phages from Thai and Vietnamese isolates, respectively, combined with the apparent broad distribution of P2-like prophage elements in *B*. *pseudomallei* highlights the success of this closely-related clade of lysogenic phages at infection and spread among a diverse spectrum of *B*. *pseudomallei* strains [16].

## Methods

### Bacterial growth and preparation of phage lysates

*Burkholderia* sp. used in this study are listed in Additional file 1. *Burkholderia* sp. and *Escherichia coli* strains were grown at 37°C with aeration in Lennox LB media as previously described [17]. For growth of *B*. *mallei*, LB was supplemented with 2-4% glycerol. Growth media for Bp82 and its derivatives were augmented with 80 μg/mL adenine [18]. All procedures involving *B*. *pseudomallei* and *B*. *mallei* were performed in Select Agent approved Biosafety Level 3 (BSL3) facilities in the Rocky Mountain Regional Biosafety Laboratory (CSU) and the United States Army Medical Research Institute of Infectious Diseases using Select Agent compliant procedures and protocols. Phage plaque plates were prepared by adding 200 μl of a *Burkholderia* sp. overnight culture to 4 mL of molten top agar (0.6% agar, 0.1% glycerol and 2 mM CaCl_2_) at 55°C followed by gentle mixing and pouring of the mixture onto LB agar plates. For the use of four-sectored 100 mL petri plates, volumes were adjusted to 100 μL of overnight culture and 2 mL molten top agar per sector. Phage lysates were either added to top agar prior to pouring onto an LB agar plate or were spotted onto solidified top agar containing bacteria and allowed to dry prior to incubation at 37°C. Phage lysates were diluted in either Phage buffer [PB; 50 mM Tris–HCl (pH 7.4), 10 mM MgSO_4_, 2 mM CaCl_2_, 75 mM NaCl] or SM buffer [50 mM Tris–HCl (pH 7.5), 100 mM NaCl, 8 mM MgSO_4_, 0.002% gelatin] [19].

### Phage isolation and enumeration

φX216 was plaque-purified twice from spontaneously formed plaques by released phage on *B*. *pseudomallei* E0237 using small scale liquid lysates using *B*. *pseudomallei* 2698a as a host strain. Plate lysates were prepared by flooding inverted plates with 5 mL of PB followed by incubation for either 3 h at 37°C or overnight at 4°C without agitation. The liquid was recovered from plates and bacteria pelleted by centrifugation at 16,000x*g* for 1 min at room temperature. Supernatants were combined and sterilized with a 0.2 μm disposable syringe filter (DISMIC-25_AS_ Life Science Products, Inc., Frederick, CO). To create adapted lysates, plate lysates were used sequentially to infect a host strain followed by lysate recovery and reinfection for two to four cycles. For liquid lysates, 1 mL of a *B*. *mallei* ATCC23344 overnight culture, 1 mL phage lysate at approximately 10^6^ pfu/mL, 1 mL 10 mM CaCl_2_ and 10 mM MgCl_2_ were combined and incubated without agitation at 37°C for 15 min for initial phage attachment. 1.5 mL each of these mixtures were inoculated into 2 × 250 mL of pre-warmed LB with 2% glycerol in two 1 L disposable fretted Erlenmeyer flasks (Corning, Elmira, NY) and incubated overnight at 37°C with aeration. After overnight incubation, lysates were sometimes treated with 1% chloroform although better results were obtained when this step was omitted. Lysates were centrifuged at 4,000x*g* for 20 min at 4°C. Supernatants were combined with 25 mL 1 M Tris–HCl (pH 7.4) to a final concentration of 50 mM Tris–HCl, pre-filtered through a 0.8 μm disposable vacuum filtration unit and then filtered through a 0.2 μm disposable vacuum filtration unit to achieve sterility (Nalgene, Rochester, NY). Lysates were stored at 4°C in the dark. To determine phage titers, lysates were serially diluted in PB and 10 μL aliquots spotted onto top agar plates with appropriate *Burkholderia* sp. tester strains. Isolated plaques were counted and titers (pfu/mL) calculated.

### Burst size determination

Phage burst sizes were determined by generation of one-step growth curves as previously described [19]. Briefly, a *B*. *mallei* ATCC23344 liquid lysate was inoculated using the same procedure described above for a single 250 mL volume. After the initial attachment mixture was incubated for 15 min and inoculated into a 1 L flask, triplicate 200 μL samples were recovered to produce T_0_ plaque plates using *B*. *mallei* ATCC23344 as the indicator strain. Triplicate samples (200 μL at 60 min, 100 μL at 80 min, and 50 μL 100 min through 180 min) were collected at 20 min intervals until 180 min post-inoculation to generate plaque plates. Plaques were counted and titers determined for each time point. One-step growth curves were repeated three times with similar results. Burst size was determined as the average fold increase in final pfu counts versus input pfu after one cycle of phage replication. Input pfu values were determined by averaging pfu/mL values taken at T_0_ and T_1_.

### Determination of phage infectivity

100 mm or four-sectored plaque plates were prepared as described above using each of the *Burkholderia* sp. strains listed in Additional file 1. Each sector was spotted with 20 μL each of *B*. *mallei* ATCC23344 liquid lysate, equating to approximately 10^6^ and 10^4^ pfu. For φ52237, sectors were additionally spotted with approximately 10^8^ pfu, a titer that was not obtained with φX216. Strains were considered positive for infection if they produced distinct plaques with either 10^6^ or 10^4^ pfu aliquots in multiple independent trials. *B*. *mallei* were considered positive for infection if plaques were observed when 10^2^ pfu were mixed with the *B*. *mallei* indicator strain in LB top agar (0.6% agar). *B*. *pseudomallei* O-antigen mutants were tested simultaneously using both spotting and mixing methods.

### Recombinant DNA techniques

DNA Restriction enzymes, T4 DNA ligase and *Taq* polymerase were purchased from NEB (Ipswich, MA) and used according to recommended protocols. Oligonucleotides were purchased from Integrated DNA Technologies (Coralville, IA) and are listed in Additional file 2. Plasmid DNA was purified using the GeneJet Plasmid Miniprep Kit from Fermentas (Glen Burnie, MD).

### PCR screening of candidate P2-like lysogens

Primer sets were designed to amplify regions that were either conserved or unique to subsets of six previously described P2-like *Burkholderia* phage genomes deposited in Genbank, (GenBank:BX571965, GenBank:BX571966, GenBank:DQ087285, GenBank:CP000623, GenBank:CP000624, GenBank:CP000085) [8]. The genomic island 2 primer set was designed to span the tRNA-Phe gene (BURPS1710b_0354) and the primers were designed to anneal to highly conserved bacterial and phage genome regions [8]. Multiplex primers were designed to have calculated T_m_ values within 1°C of one another and to amplify products separated in size by approximately 100 bp. Purified bacterial genomic DNA was used as a PCR template.

### Lysogen isolation

A top agar plate of the *B*. *pseudomallei* 1710b derivative Bp516 was spotted with approximately 10^6^ pfu/mL of 1710b-adapted φX216 plate lysate [20]. Bacteria were recovered from turbid zones of lysis and streaked to isolation. Isolated colonies were assessed for φX216 infectability and screened by PCR for the presence of the φX216 prophage at genomic island 2 and with other φX216 primer sets.

### *B*. *pseudomallei* O-antigen mutant strain construction

DNA fragments corresponding to the 470-bp 5’ and 608-bp 3’ regions of the *wbiE* gene of Bp1026b were PCR amplified from genomic DNA using *Taq* polymerase with primers P2348 & P2349 and P2350 & P2351, respectively, and joined by overlap extension PCR [21]. The resulting 1,068-bp product was digested with *Eco*RI and ligated with *Eco*RI digested pEXGm5B [20] DNA to yield pPS2882. The 1.4-kb *FRT*-Km^r^*FRT* cassette of pFKm4 [20] as released by digestion with *Xma*I and ligated between the partially *Xma*I-digested chromosomal DNA fragments contained in pPS2882 to create pPS2896. The pPS2896 plasmid was used to delete the *wbiE* region from Bp82 by allelic exchange employing previous published procedures [20,22]. This yielded the Δ*wbiE* mutant Bp82.39 and the presence of the correct mutant allele was confirmed by PCR amplification of the deletion region using primers P2368 and P2369. Sequence-defined *B*. *pseudomallei* 1026 *wbi*::T24 transposon insertion mutants were obtained through an ongoing project.

### Genomic DNA purification

Bacterial genomic DNA was purified with the Qiagen Gentra Puregene Gram negative Bacteria kit according to the manufacturer’s recommendations (Qiagen, Valencia, CA). Phage particles were semi-purified by polyethylene glycol precipitation as previously described [23]. Briefly, 30 g NaCl was added to 500 mL of sterile filtered *B*. *mallei* ATCC23344 liquid lysate (10^8^ pfu/mL) and stirred continuously on ice while 50 g of polyethylene glycol 8000 (PEG) was slowly added. The mixture was then stirred continuously overnight at 4°C. PEG-precipitated lysates were pelleted by centrifugation at 11,000x*g* for 15 min at 4°C and the supernatant discarded. Pellets were suspended in 8 mL SM buffer, combined with 8 mL chloroform, vortexed vigorously for 30 s and centrifuged at 4,000x*g* for 15 min at 4°C. Aqueous layers were retained and extracted two additional times with chloroform to remove any remaining PEG. This concentrated phage particles approximately 10-fold. Phage DNA was purified using a modification of the protocol described by Kaslow [24]. To 3 mL total concentrated lysate, 15 μL DNase I (1 mg/mL) and 30 μL RNase A (10 mg/mL) were added and incubated at 37°C for 30 min. Then 150 μL 10% SDS, 125 μL 0.5 M EDTA (pH 8.0), and 250 μL STEP buffer [0.1% SDS, 10 mM Tris–HCl (pH 7.4), 80 mM EDTA, 1 mg/mL proteinase K] were added, and the mixture incubated for 30 min at 65°C. Genomic DNA from enzymatically treated lysates was phenol + chloroform extracted. 3.5 mL TE - saturated phenol was added to enzymatically treated lysates, mixed by inversion, and centrifuged at 800x*g* for 5 min at room temperature. The aqueous phase was retained and extracted twice with 3.5 mL phenol + chloroform (1:1) and once with 3.5 mL chloroform. Phage genomic DNA was ethanol precipitated by adding 1.2 mL 7.5 M NH_4_-acetate and 4.5 mL −20°C Ethanol (96%), followed by 15 min incubation on ice. Phage genomic DNA was spooled onto a sealed Pasteur pipette, transferred to a fresh 1.5 mL microfuge tube, air dried briefly and suspended in 200 μL TE buffer resulting in a DNA concentration of approximately 1 μg/μL.

### Sequencing and annotation

Random and φ52237-sequence guided φX216 genome fragment clones were constructed by restriction digest of purified φX216 genomic DNA with *Eco*RI, *Eco*RI + *Hin*dIII or *Age*I and ligation with *Eco*RI, *Eco*RI + *Hin*dIII or *Sma*I digested pUC19 DNA [25], respectively, followed by transformation of *E*. *coli* DH5α or GBE180 [26] using standard transformation protocols [27] and recovery of white colonies on LB plates containing 100 μg/mL ampicillin and 50 μg/mL 5’-bromo-4-chloro-3-indolyl-β-D-galactopyranoside (X-gal). φ52237-sequence-guided PCR amplicons were designed to close gaps and confirm fragment clone borders. Sequencing was accomplished using M13F and M13R primers, as well as φ52237-sequence guided primer walking of fragment clones and PCR amplicons using an ABI 3130xL Genetic Analyzer (Applied Biosystems, Carlsbad, CA) at the Colorado State University Proteomics and Metabolomics Facility. φX216 Illumina sequencing libraries were prepared using the TruSeq DNA Sample Preparation Kit v2, (Illumina, San Diego, CA), following the manufacturer's instructions. Phage DNA was fragmented to a range of 300–400 bp using a Covaris acoustic shearing device, (Covaris Inc., Woburn, MA) followed by 3' adenylation and adapter ligation. Ligation products were purified on an agarose gel and the DNA fragments enriched via PCR. Fragmented Phage DNA was sequenced by high-throughput Illumina parallel sequencing using 100 bp mate-pair Illumina HiSeq 2000 reversible terminator chemistry. The library was run on 15% of a single lane. Reads were trimmed for quality and *de novo* short-read genome assembly was performed using the Velvet 1.1.05 sequence assembler algorithms with a hash length of 99 and a final graph with 3 nodes and n50 of 37412 nt [28]. Open reading frames were identified with GeneMark gene prediction software using a viral-optimized Heuristic approach [29]. Putative gene identification was conducted by sequence alignment with φ52237 (GenBank:DQ087285.2) [8] and individual open reading frames queried using the NCBI Basic Alignment Search Tool (BLAST). Genome annotation, mapping, sequence alignments, and comparative analyses were conducted using Gene Construction Kit v3.0 and Geneious Pro 5.4.6 bioinformatics software. The annotation map was created using Adobe Illustrator CS5. The final φX216 genome sequence has been deposited in GenBank under accession # JX681814.

## Competing interests

The authors declare no competing interests.

## Authors’ contributions

**BHK**, **CRC**, **DD**, **KV**, and **HPS** conceived and designed the experiments. **BHK** conducted experiments with *B*. *pseudomallei* and other *Burkholderia* strains. **DD** conducted host range tests with *B*. *mallei* strains. **BHK**, **CRC** and **SLJ** conducted genome sequencing and annotation. **BHK**, **CRC**, **DD**, and **HPS** wrote the manuscript. All authors read and approved the final manuscript.

## Supplementary Material

Additional file 1 **φX216 host range, word document, Host range of φX216.** Table of φX216 host range for 72 *B. pseudomallei* strains and other *Burkholderia* species. Click here for file

Additional file 2 **Oligonucleotides, word document, Oligonucleotides and probe regions.** Table of oligonucleotides and probe regions designed for this study. Click here for file
