# Supplementary material for: φX216, a P2-like bacteriophage with broad Burkholderia pseudomallei and B. mallei strain infectivity
Source: BMC Microbiol. 2012 Dec 7;12:289. doi: 10.1186/1471-2180-12-289 (PMC3548686; doi:10.1186/1471-2180-12-289)
Supplement: Additional file 1 — φX216 host range, word document, Host range of φX216. Table of φX216 host range for 72 B. pseudomallei strains and other Burkholderia species. [file 1471-2180-12-289-S1.docx]

**Additional file 1. Host range of φX216**

| ***Burkholderia* sp.** | **Strain**  **(Notes)** | **φX216**  **infectivity^a^** | **φ52237**  **infectivity^a^** | **Strain source** |
| --- | --- | --- | --- | --- |
| *B. pseudomallei* | **Clinical**  **Isolates** | 21/30 (70.0%) | 21/30 (70.0%) |  |
|  | 2613a | + | + | [[1](#_ENREF_1)] |
|  | 2618a | + | + | [[1](#_ENREF_1)] |
|  | 2625a | + | + | [[1](#_ENREF_1)] |
|  | 2637a | + | + | [[1](#_ENREF_1)] |
|  | 2650a | + | + | [[1](#_ENREF_1)] |
|  | 2660a | + | + | [[1](#_ENREF_1)] |
|  | 2661a | + | + | [[1](#_ENREF_1)] |
|  | 2667a | + | + | [[1](#_ENREF_1)] |
|  | 2668a | + | + | [[1](#_ENREF_1)] |
|  | 2670a | + | + | [[1](#_ENREF_1)] |
|  | 2673a | + | + | [[1](#_ENREF_1)] |
|  | 2682a | + | + | [[1](#_ENREF_1)] |
|  | 2685a | + | + | [[1](#_ENREF_1)] |
|  | 2692a | + | + | [[1](#_ENREF_1)] |
|  | 2698a | + | + | [[1](#_ENREF_1)] |
|  | 2704a | + | + | [[1](#_ENREF_1)] |
|  | 2708a | + | + | [[1](#_ENREF_1)] |
|  | 2717a | + | + | [[1](#_ENREF_1)] |
|  | 2719a | + | + | [[1](#_ENREF_1)] |
|  | 2764b | + | + | S. Peacock |
|  | 2769a | + | + | [[1](#_ENREF_1)] |
|  | 2614a | - | - | [[1](#_ENREF_1)] |
|  | 2617a | ­- | ­- | [[1](#_ENREF_1)] |
|  | 2640a | - | - | [[1](#_ENREF_1)] |
|  | 2665a | - | - ^b^ | [[1](#_ENREF_1)] |
|  | 2671a | - | - | [[1](#_ENREF_1)] |
|  | 2674a | - | - | [[1](#_ENREF_1)] |
|  | 2677a | - | - | [[1](#_ENREF_1)] |
|  | 2689b | - | - | [[1](#_ENREF_1)] |
|  | 2694a | - | - | [[1](#_ENREF_1)] |
|  |  |  |  |  |
| *B. pseudomallei* | **Environmental**  **Isolates** | 26/30 (86.7%) | 26/30 (86.7%) |  |
|  | E0016 | + | + | [[1](#_ENREF_1)] |
|  | E0021 | + | + | [[1](#_ENREF_1)] |
|  | E0024 | + | + | [[1](#_ENREF_1)] |
|  | E0031 | + | + | [[1](#_ENREF_1)] |
|  | E0034 | + | + | [[1](#_ENREF_1)] |
|  | E0037 | + | + | [[1](#_ENREF_1)] |
|  | E0181 | + | + | [[1](#_ENREF_1)] |
|  | E0235 | + | + | [[1](#_ENREF_1)] |
|  | E0237  (φX216 source strain) | + | + | [[1](#_ENREF_1)] |
|  | E0241 | + | + | [[1](#_ENREF_1)] |
|  | E0279 | + | + | [[1](#_ENREF_1)] |
|  | E0342 | + | + | [[1](#_ENREF_1)] |
|  | E0345 | + | + | [[1](#_ENREF_1)] |
|  | E0356 | + | + | [[1](#_ENREF_1)] |
|  | E0366 | + | + | [[1](#_ENREF_1)] |
|  | E0371 | + | + | [[1](#_ENREF_1)] |
|  | E0372 | + | + | [[1](#_ENREF_1)] |
|  | E0377 | + | + | [[1](#_ENREF_1)] |
|  | E0378 | + | + | [[1](#_ENREF_1)] |
|  | E0380 | + | + | S. Peacock |
|  | E0383 | + | + | [[1](#_ENREF_1)] |
|  | E0384 | + | + | [[1](#_ENREF_1)] |
|  | E0386 | + | + | S. Peacock |
|  | E0393 | + | + | [[1](#_ENREF_1)] |
|  | E0394 | + | + | [[1](#_ENREF_1)] |
|  | E0411 | + | + | [[1](#_ENREF_1)] |
|  | E0008 | - | - ^b^ | [[1](#_ENREF_1)] |
|  | E0183 | - | - ^b^ | [[1](#_ENREF_1)] |
|  | E0350 | - | - | S. Peacock |
|  | E0396 | - | - | [[1](#_ENREF_1)] |
|  |  |  |  |  |
| *B. pseudomallei* | **Other Isolates** | 9/12 (75%) | 9/12 (75%) |  |
|  | 1710a | + | + | [[2](#_ENREF_2)] |
|  | 1710b | + | + | [[2](#_ENREF_2)] |
|  | 708a | + | + | [[3](#_ENREF_3)] |
|  | MSHR305 | + | + | [[1](#_ENREF_1)] |
|  | K96243 | + | + | [[4](#_ENREF_4)] |
|  | S13 | + | + | [[5](#_ENREF_5)] |
|  | 406e | + | + | [[5](#_ENREF_5)] |
|  | 1026b | + | + | [[6](#_ENREF_6)] |
|  | Bp82  (1026b ∆*purM*) | + | + | [[7](#_ENREF_7)] |
|  | 1106b | + | + | [[2](#_ENREF_2)] |
|  | 1106a | - | - | [[2](#_ENREF_2)] |
|  | MSHR668 | - | - ^b^ | [[1](#_ENREF_1)] |
|  | Pasteur 6068 | - | - | [[1](#_ENREF_1)] |
|  | Bp523  (1710b-derivative  φX216 lysogen) | - | - | This study |
|  |  |  |  |  |
| *B. pseudomallei* | **O-antigen mutants** |  |  |  |
|  | q0035h12  (1026b *wbiA*::T24) | + | + | This study |
|  | q0028a9  (1026b *wbiB*::T24) | + | + | This study |
|  | q0010e11  (1026b *wbiC*::T24) | + | + | This study |
|  | q0077c2  (1026b *wbiD*::T24) | + | + | This study |
|  | q0058o8  (1026b *wbiE*::T24) | + | + | This study |
|  | q0107d23  (1026b *wbiF*::T24) | + | + | This study |
|  | q0001n23  (1026b *wbiF*::T24) | + | + | This study |
|  | q0098b10  (1026b *wbiG*::T24) | + | + | This study |
|  | q0107i8  (1026b *wbiH*::T24) | + | + | This study |
|  | q0119k15  (1026b *wbiI*::T24) | + | + | This study |
|  | Bp82.39  (1026b ∆*purM* ∆*wbiE)* | + | + | This study |
|  |  |  |  |  |
| *B. mallei* | ATCC 23344 | + | + | [[8](#_ENREF_8)] |
|  | NCTC 10248 | + | + | [[9](#_ENREF_9)] |
|  | NCTC 10229 | + | + | [[9](#_ENREF_9)] |
|  | NCTC 10260 | + | + | [[9](#_ENREF_9)] |
|  | NCTC 10247 | + | + | [[9](#_ENREF_9)] |
|  | NCTC 3708 | + | + | [[9](#_ENREF_9)] |
|  | NCTC 3709 | + | + | [[9](#_ENREF_9)] |
|  | ATCC 10399 | + | + | [[9](#_ENREF_9)] |
|  | ATCC 15310 | + | + | [[9](#_ENREF_9)] |
|  |  |  |  |  |
| *B.mallei* | **O-antigen mutants** |  |  |  |
|  | DB110795 (*wbiG*::IS) | - | - | [[9](#_ENREF_9)] |
|  | ISU (O-antigen^-^) | - | - | [[9](#_ENREF_9)] |
|  | NCTC 120 (*wbiE*::IS) | - | - | [[9](#_ENREF_9)] |
|  | NCTC 120 pBHR1-*wbiE* | - | - | [[9](#_ENREF_9)] |
|  |  |  |  |  |
| *B. gladioli* pv. *cocovenenans* | ATCC 33664 | - | - | [[10](#_ENREF_10)] |
|  |  |  |  |  |
| *B. oklahomensis* | C6786 | - | - | [[11](#_ENREF_11)] |
|  |  |  |  |  |
| *B. thailandensis* | E264 | - | - | [[12](#_ENREF_12)] |
|  | E421 | - | - | [[1](#_ENREF_1)] |
|  | E426 | - | - | [[1](#_ENREF_1)] |
|  |  |  |  |  |
| *B. ubonensis* | 184 | - | - | [[13](#_ENREF_13)] |
|  | A20 | - | - | [[14](#_ENREF_14)] |
|  | A1301 | - | - | [[13](#_ENREF_13)] |
|  |  |  |  |  |
| *B. vietnamensis* | H4102 | - | - | [A.](#_ENREF_37) Hoffmaster, |
|  |  |  |  |  |

^a^+ indicates plaque formation when spotted with < 10^6^ pfu φX216 or φ52237 except for *mallei* strains where + indicates plaque formation when mixed with approximately 10^2^ pfu φX216 or φ52237

^b^Plaque formation when spotted with >10^8^ pfu φ52237 but not < 10^6^ pfu.

1. Tuanyok A, Stone JK, Mayo M, Kaestli M, Gruendike J, Georgia S, Warrington S, Mullins T, Allender CJ, Wagner DM *et al*: **The genetic and molecular basis of O-antigenic diversity in *Burkholderia pseudomallei* lipopolysaccharide**. *PLoS Negl Trop Dis* 2012, **6**(1):e1453.

2. Nandi T, Ong C, Singh AP, Boddey J, Atkins T, Sarkar-Tyson M, Essex-Lopresti AE, Chua HH, Pearson T, Kreisberg JF *et al*: **A genomic survey of positive selection in *Burkholderia pseudomallei* provides insights into the evolution of accidental virulence**. *PLoS Pathog* 2010, **6**(4):e1000845.

3. Trunck LA, Propst KL, Wuthiekanun V, Tuanyok A, Beckstrom-Sternberg SM, Beckstrom-Sternberg JS, Peacock SJ, Keim P, Dow SW, Schweizer HP: **Molecular basis of rare aminoglycoside susceptibility and pathogenesis of *Burkholderia pseudomallei* clinical isolates from Thailand**. *PLoS Negl Trop Dis* 2009, **3**(9):e519.

4. Holden MT, Titball RW, Peacock SJ, Cerdeno-Tarraga AM, Atkins T, Crossman LC, Pitt T, Churcher C, Mungall K, Bentley SD *et al*: **Genomic plasticity of the causative agent of melioidosis, *Burkholderia pseudomallei***. *Proc Natl Acad Sci U S A* 2004, **101**(39):14240-14245.

5. Tumapa S, Holden MT, Vesaratchavest M, Wuthiekanun V, Limmathurotsakul D, Chierakul W, Feil EJ, Currie BJ, Day NP, Nierman WC *et al*: ***Burkholderia pseudomallei* genome plasticity associated with genomic island variation**. *BMC Genomics* 2008, **9**:190.

6. Hayden HS, Lim R, Brittnacher MJ, Sims EH, Ramage ER, Fong C, Wu Z, Crist E, Chang J, Zhou Y *et al*: **Evolution of *Burkholderia pseudomallei* in recurrent melioidosis**. *PLoS One* 2012, **7**(5):e36507.

7. Propst KL, Mima T, Choi KH, Dow SW, Schweizer HP: **A *Burkholderia pseudomallei* D*purM* mutant is avirulent in immunocompetent and immunodeficient animals: candidate strain for exclusion from select-agent lists**. *Infect Immun* 2010, **78**(7):3136-3143.

8. Nierman WC, DeShazer D, Kim HS, Tettelin H, Nelson KE, Feldblyum T, Ulrich RL, Ronning CM, Brinkac LM, Daugherty SC *et al*: **Structural flexibility in the *Burkholderia mallei* genome**. *Proc Natl Acad Sci U S A* 2004, **101**(39):14246-14251.

9. Woods DE, Jeddeloh JA, Fritz DL, DeShazer D: ***Burkholderia thailandensis* E125 harbors a temperate bacteriophage specific for *Burkholderia mallei***. *J Bacteriol* 2002, **184**(14):4003-4017.

10. Somprasong N, McMillan I, Karkhoff-Schweizer RR, Mongkolsuk S, Schweizer HP: **Methods for genetic manipulation of *Burkholderia gladioli* pathovar *cocovenenans***. *BMC Res Notes* 2010, **3**:308.

11. Glass MB, Steigerwalt AG, Jordan JG, Wilkins PP, Gee JE: ***Burkholderia oklahomensis* sp. nov., a *Burkholderia pseudomallei*-like species formerly known as the Oklahoma strain of *Pseudomonas pseudomallei***. *Int J Syst Evol Microbiol* 2006, **56**(Pt 9):2171-2176.

12. Kim HS, Schell MA, Yu Y, Ulrich RL, Sarria SH, Nierman WC, DeShazer D: **Bacterial genome adaptation to niches: divergence of the potential virulence genes in three *Burkholderia* species of different survival strategies**. *BMC Genomics* 2005, **6**:174.

13. Levy A, Merritt AJ, Aravena-Roman M, Hodge MM, Inglis TJ: **Expanded range of *Burkholderia* species in Australia**. *Am J Trop Med Hyg* 2008, **78**(4):599-604.

14. Marshall K, Shakya S, Greenhill AR, Padill G, Baker A, Warner JM: **Antibiosis of *Burkholderia ubonensis* againist *Burkholderia pseudomallei*, the causative agent for melioidosis**. *Southeast Asian J Trop Med Public Health* 2010, **41**(4):904-912.
