# Supplementary material for: φX216, a P2-like bacteriophage with broad Burkholderia pseudomallei and B. mallei strain infectivity
Source: BMC Microbiol. 2012 Dec 7;12:289. doi: 10.1186/1471-2180-12-289 (PMC3548686; doi:10.1186/1471-2180-12-289)
Supplement: Additional file 2 — Oligonucleotides, word document, Oligonucleotides and probe regions. Table of oligonucleotides and probe regions designed for this study. [file 1471-2180-12-289-S2.docx]

**Additional file 2. Oligonucleotides and probe regions**

| **Primer number** | **Sequence**  **5’→3’** | **Probe** | **Gene region**  **(amplified fragment size)** |
| --- | --- | --- | --- |
| 2458 | AGCTGCTGCACGACAAGTATTTCC | P2-like 1 | N capsid  (248 bp) |
| 2459 | ACGATTCGTAGTTCTCGATGCGGT |  |  |
| 2460 | ATGCTCATGTCCCTCGACCAATTC | P2-like 2 | Fels-2  (418 bp) |
| 2461 | TTGTCCTTGCGCGTGTTCTTCT |  |  |
| 2462 | ACCATCATTCCATGAGTTGCGCTG | P2-like group A | integrase  (316 bp) |
| 2463 | TTGTCTAATTCGCGAGCCGTCGAT |  |  |
| 2464 | TCGCTTGAGTAGTGATCCGCGAAA | φX216 scrnA | gene #46  (433 bp) |
| 2465 | GTTGCCATGGTCCATCACTTGCAT |  |  |
| 2466 | GCAATCAAACCCATGTGCTCGTCA | φX216 scrnB | Between genes  #30 and #31  (284 bp) |
| 2467 | TGATGTTTCCGCAAGCAGCTCATC |  |  |
| 2468 | CACCgaattcAGCGCGCGTACTTCGAATATCA | GI2 | tRNA-Phe/integrase  (578 bp) |
| 2469 | GAAaagcttGCATCTACAACCACGCATGGCAAA |  |  |
| 2348 | CACCGAATTCGCGTGCGCAAACTCAGGTCATAAT |  | Δ*wbiE*-5’  (470 bp) |
| 2349 | TAATCCCGGGTAACGGCCACGGTAATGTGGGTTA |  |  |
| 2350 | GTGGCCGTTACCCGGGATTAAGTTCATCGTCGTCCAGAATCGCA |  | Δ*wbiE*-3’  (608 bp) |
| 2351 | CACCGAATTCTCTCGATCACACGCACGTCATTCA |  |  |
| 2368 | ATATCGCGAACAGGCGAACATGAC |  | Δwbi-chk-F |
| 2369 | GTCGCAATGTGGCGTCGATTTCAA |  | Δwbi-chk-R |
